# Perceptions of e-cigarettes among smokers and non-smokers in households with children in rural China: A cross-sectional study

**DOI:** 10.18332/tid/133264

**Published:** 2021-04-09

**Authors:** Duan Zhao, Abu S. Abdullah, Tong Wen, Xiaoxiao Chen, Xia Xiao, Zixian Pan, Jingyi He, Dilshat S. Urmi, Wei Hao, Haijiang Lin, Pinpin Zheng

**Affiliations:** 1 Global Health Research Center, Duke Kunshan University, Kunshan, China; 2 Duke Global Health Institute, Duke University, Durham, United States; 3 Boston Medical Center, Boston University School of Medicine, Boston, United States; 4 Taizhou City Centers for Disease Control and Prevention, Taizhou, China; 5 School of Public Health, Kunming Medical University, Kunming, China; 6 Department of Preventive Medicine, School of Public Health, Fudan University, Shanghai, China

**Keywords:** e-cigarettes, perception, tobacco policy, rural, China

## Abstract

**INTRODUCTION:**

The perceived health benefits and effectiveness of electronic cigarettes (e-cigarettes) in quitting smoking may affect e-cigarette usage, however, research on the use of e-cigarettes among the Chinese, especially among the rural Chinse, is scarce. This study examined factors associated with perceptions of e-cigarette related harms, benefits, and addictiveness, among smoker and non-smoker households with children in rural China, to support the design of population-based interventions targeting rural Chinese households.

**METHODS:**

In a cross-sectional study design, using a structured questionnaire, we collected data from the household members of children in two selected rural communities in China. Descriptive analyses were used to characterize respondents; χ^2^ test and Fisher’s exact probability test were used to compare the perceptions of e-cigarettes between different sociodemographic groups. Logistic regression was used to determine predictors for e-cigarette harms, benefits, and addictiveness, adjusting for demographic and other characteristics.

**RESULTS:**

The overall participation rate was 81% (1211/1498). Of the participants, 668 (55%) were smokers and 543 (45%) were non-smokers; 53% knew about e-cigarettes. Participants from rural Dali (77% vs 59%), those who were ethnic minority (76% vs 59%), those who perceived increased COPD risks from smoking (mean score 4.37 vs 4.18) and concerned about harmful effects of secondhand smoke (SHS) exposure to children (mean score 4.48 vs 4.30) and adults (mean score 4.06 vs 3.87) were more likely to believe that e-cigarettes were less harmful (p<0.05). Participants with more knowledge about the harm of smoking were more likely to believe that e-cigarettes were helpful in quitting smoking (p<0.05). Of those participants who knew about e-cigarettes, females (19%) were significantly more likely to believe that e-cigarettes are addictive than males (10%). In the logistic regression analyses, believing e-cigarettes are helpful to quit smoking was the only variable associated with holding a higher knowledge about smoking and SHS exposure (OR=0.608; 95% CI: 0.450–0.820).

**CONCLUSIONS:**

Our results showed that more than half of the rural household members who have a child at home were aware of e-cigarettes. Knowledge about health impacts of SHS exposure and perceptions about the benefits, harms and addictiveness of e-cigarette use varied among the participants, with a significant proportion of participants having wrong information. Public health campaigns to disseminate evidence-based information of e-cigarette benefits and harms are warranted. As knowledge about the harmfulness of smoking and SHS exposure was associated with perceived e-cigarette benefits, particular focus should be given to increasing knowledge about the health hazards related to smoking and SHS exposure.

## INTRODUCTION

Electronic cigarettes (e-cigarettes), also known as electronic nicotine delivery systems (ENDS), are believed to be less harmful to health than combustible cigarettes and purported to be a safer alternative for conventional smokers and a tool for smoking cessation^1^. However, possible public health benefits can only be achieved if e-cigarette users do not switch to or concurrently use combustible cigarettes^2^. In addition, the long-term effects of e-cigarettes on health and their use as smoking cession aids are inconclusive^3^. Some studies have found e-cigarette use increased cessation rates^4-6^, but other studies have found contradictory results^7,8^. Despite the inconclusive results about the benefits of e-cigarettes, their use has been increasing in recent years in China and around the world^9,10^. Meanwhile, heated tobacco products (HTPs such as IQOS ‘I quit original smoking’) have recently been reintroduced to the mass market. While HTPs are classified as tobacco products in some countries such as the US, they are marketed as a variation of e-cigarettes in other countries such as Korea. In China, both HTPs and heated liquid e-cigarettes are referred to as ‘dian zi yan’ (i.e. e-cigarettes) by sellers and consumers. A significant proportion of participants in an earlier study among the Chinese did not possess appropriate knowledge about e-cigarettes^11^ and many even never heard of e-cigarettes^12^. Therefore, a deeper understanding of the awareness and perception of e-cigarette use at the population level is critical to developing any preventive measures.

Perceptions of risk can affect tobacco use^13^, similarly, the perceived health benefits and the effectiveness of e-cigarettes in quitting may affect their usage. Awareness of e-cigarettes was found to be most common among smokers and was related to being educated and being younger^14^. Female and better-educated smokers were more likely to use e-cigarettes^15^. In earlier studies among adolescents and young adults, the main reasons to use e-cigarettes were curiosity, attractiveness and appeal of flavors and cleanliness, that they were fashionable, had harm reduction potential, and an aid to quit smoking. However, most of the published studies were conducted in high-income countries with limited studies conducted in developing countries. In China, several recent studies have focused on the urban population in China^9,10,16,17^, with no studies focusing on e-cigarette use perception among adults in households of rural China.

The number of smokers who know and have tried e-cigarettes is growing fast in China. For example, in the International Tobacco Control Policy Evaluation Project (ITC)-China^18^, the percentage of smokers who knew of e-cigarettes rose from 29% to 60% and those who had tried e-cigarettes rose from 2% to 11%, between 2009 and 2014. This increasing trend in e-cigarette awareness and use may have a negative impact on the overall tobacco control effort in China and underscore the need for public health measures addressing e-cigarette use. Although the use of e-cigarettes has been increasing in China, the governance of e-cigarette use has been loose compared to other countries that have enacted relatively strict regulations for e-cigarette use and supply^19^. Until November 2019, the State Tobacco Monopoly Administration of China and the State Administration for Market Regulation issued the ‘Notice on Further Protecting Minors from E-cigarettes’ and banned all e-cigarette sales online^20^. Understanding public perceptions can inform regulation, prevention messaging and other public health actions for e-cigarette use in China and in other low- and middle-income countries. Also, targeting rural populations is unique in that many of the available tobacco control programs and services are enforced in urban areas while their availability in rural settings is scarce, making rural populations vulnerable to receiving wrong information (i.e. misinformation about e-cigarette benefits) and, thus, engaging in risky behaviors^18^. Also, as part of our ongoing research on family-based smoking cessation promotion and secondhand smoke exposure reduction interventions, we have found that children aged ≤5 years are vulnerable to secondhand smoke exposure in the household. Our exploratory work in urban China made us realize that certain groups of rural populations (i.e. friends of urban e-cigarette users) use e-cigarettes that are provided by their urban friends^21^. Given the growing e-cigarette market in China and the lack of tobacco control measures in rural China^9^, we examined, in the present study, the e-cigarette use perceptions among the rural population. Rather than designing a new study, we utilized the sampling framework of an existing study that was conducted among household members of children aged ≤5 years. Therefore, this study examined how smoking status and sociodemographic factors were associated with perceptions of e-cigarette related harms, benefits, and addictiveness, among the adults in households with children in rural China.

## METHODS

### Settings

The study settings were rural communities in Taizhou and Dali. In these two communities, villages were purposively selected, to cover diverse sociocultural aspects of the local population, as recommended by the local collaborators. Taizhou is located in central Zhejiang province, in the eastern part of China. Taizhou is an economically developed region and the GDP per capita of Taizhou was RMB 72912 (Chinese Renminbi, about US$10000) in 2017^22^. The rural population accounted for 37.8% of the total population (about 6 million) of Taizhou^22^. Four villages were purposively selected for this study, including two in Luqiao district and two in Linhai city in Taizhou.

Dali is the autonomous prefecture for the Bai minority, located in the western part of China. The GDP per capita in 2017 of Dali was RMB 29846 (about US$4500)^23^. The four villages from Jianchuan country in Dali were purposively selected for this study. The total population of Jianchuan was over 18 million in 2016 and the rural population accounted for 27% of the total population^24^.

### Sample

The study population consisted of household members of children aged ≤5 years in the selected community. To be eligible, all households needed to have at least an adult (i.e. aged ≥18 years) smoker (who smoked one or more cigarettes daily for the past 30 days) who resided in the same house with the child and was able to communicate in Mandarin Chinese. All the eligible households were approached to participate in the study. These households were part of another large ongoing study addressing secondhand smoke exposure among children. One smoker and the main caregiver of the child were invited to participate in the survey. If a household contained more than one smoker, then the smoker who spent more time with the child was recruited. The study was approved by the ethical review board at Duke Kunshan University (IRB No: 2016ABDU003). All participants consented to participate in our survey. The data used and analyzed in this study are available from the corresponding author on reasonable request.

### Procedure

Local community health workers (CHWs) or village doctors screened households, following the child health records available in the community health center, and invited eligible households to participate in the study. Trained interviewers, accompanied by CHWs, visited households that agreed to participate in the study during an agreed time. The interviewers described the purpose and details of the study and sought informed consent from the participants. After confirming the subjects’ informed consent, interviewers completed the survey interview.

### Data collection

The study was conducted in March 2018 in Taizhou and June 2018 in Dali, separately. Students from local colleges/universities were recruited as interviewers to collect data. Interviewers received 3 hours training to learn the necessary communication and interviewing skills. The researcher took the interviewers through each questionnaire question and explained thoroughly the information required. Practice interview sessions were organized to allow interviewers to learn the skills.

A structured Chinese questionnaire, developed with reference to the available literature, was used for data collection and was pilot tested among ten potential subjects before finalization. The interviewers completed the survey via home visits, accompanied by a local CHW/village doctor. After giving informed consent, each household answered a questionnaire containing two parts: Part I for smokers and Part II for caregivers. If the smoker was the same person as the caregiver, then he/she only needed to answer the questions about the children’s health in Part II. The questionnaire was: 1) filled in by either the subjects themselves or 2) the subjects responded to the questions orally and the interviewers wrote down the answers on their behalf.

### Measures

The two-part questionnaire (Part I for smokers and Part II for caregivers) included the following domains.

#### Sociodemographic characteristics

These included region, type of participants (smoker or caregiver), gender, age, educational level, ethnicity, marital status, and occupation (Table 1).

**Table 1:** Demographic characteristics of participants by smoking status, rural areas of Taizhou and Dali, China 2018 (N=1211)*

*Demographics*	*Overall n (%)*	*Smoker n (%)*	*Nonsmoker n (%)*	*p*
**Total**	1211	668	543	
**Region** (n=1211)				0.0001
Taizhou	891 (74)	462 (69)	429 (79)	
Dali	320 (26)	206 (31)	114 (21)	
**Age** (years) (n=1141)				0.7499
≤34	413 (36)	234 (36)	179 (37)	
35–54	385 (34)	227 (35)	158 (32)	
≥55	343 (30)	194 (29)	149 (31)	
**Gender** (n=1168)				<0.0001
Male	676 (58)	659 (99)	17 (3)	
Female	492 (42)	6 (1)	486 (97)	
**Ethnicity** (n=1165)				<0.0001
Chinese Han	903 (78)	474 (72)	429 (85)	
Minority	262 (22)	188 (28)	74 (15)	
**Marital status** (n=1165)				0.0622
Married	1143 (98)	652 (98)	491 (97)	
Single	12 (1)	7 (1)	5 (1)	
Divorced or widowed	10 (1)	2 (1)	8 (2)	
**Occupation** (n=1164)				<0.0001
Farmer	397 (34)	233 (35)	164 (33)	
Self-employed	182 (16)	138 (21)	44 (9)	
Unemployed	206 (18)	41 (6)	165 (33)	
Worker	247 (21)	177 (27)	70 (14)	
Other	132 (11)	74 (11)	58 (11)	
**Education level** (n=1157)				<0.0001
Primary school or below	335 (29)	152 (23)	183 (36)	
Middle school	476 (41)	307 (47)	169 (34)	
High school or above	346 (30)	197 (30)	149 (30)	

* N may not equal to 1211 for some variables due to missing values.

#### Knowledge about smoking and secondhand smoke (SHS) exposure

The first four items (Table 2) were measured using a 5-point Likert scale with response categories ranging from 1 (minimum risk) to 5 (maximum risk). The last four items were measured using a 5-point Likert scale with response categories ranging from 1 (totally disagree) to 5 (totally agree) with those who responded ‘I don't know’ coded as zero. Higher scores indicated a higher perceived risk on each item. The mean total score for the eight items was calculated to evaluate overall smoking-related risk perception. Smoking-related risk perception combines perception about dangers of smoking and perception about dangers of SHS.

**Table 2:** Smoking and secondhand smoke (SHS) exposure related knowledge among participants by smoking status, rural areas of Taizhou and Dali, China 2018 (N=1211)

*Variables*	*Overall mean±SD*	*Smoker mean±SD*	*Non-smoker mean±SD*	p*
Smoking increases the risk of lung cancer	4.12±1.13	4.07±1.16	4.19±1.09	0.0746
Smoking increases the risk of COPD	4.05±1.13	4.00±1.16	4.12±1.08	0.0894
Smoking increases the risk of heart diseases	3.74±1.27	3.65±1.34	3.85±1.18	**0.0069**
Concerned about the harmful effects of SHS exposure to children’s health	4.29±1.06	4.19±1.13	4.41±0.95	**0.0003**
SHS exposure from smokers can cause lung cancer in non-smokers	3.05±1.62	2.93±1.68	3.21±1.52	**0.0003**
SHS exposure from smoker is harmful to infant and children’s health	3.98±1.17	3.91±1.19	4.06±1.13	**0.0262**
SHS exposure from smoker is harmful to adults’ health	3.75±1.23	3.67±1.27	3.85±1.15	**0.0093**
Smoking around your children would cause adverse health effects among children	3.98±1.16	3.91±1.20	4.07±1.11	**0.0213**
Mean total score of combined smoking and SHS exposure related knowledge	3.89±0.90	3.82±0.93	3.97±0.86	**0.0039**

* p for Student’s t-tests.

#### Awareness of e-cigarettes

This was assessed by asking a single-item question, ‘Have you ever heard of electronic cigarettes prior to this survey?’ with a brief description of e-cigarettes and a picture showing different types of e-cigarettes (with a ‘yes/no’ forced choice format response).

#### Perceptions of e-cigarettes

These were measured by asking three items including perceptions of e-cigarette harmfulness, benefits, and addictiveness, relative to traditional cigarettes. Questions asked included, ‘How harmful do you think the e-cigarettes are compared to combustible cigarettes?’ with response categories of ‘less harmful’, ‘equally harmful’, and ‘more harmful’. Benefits were measured by asking, ‘Do you believe that e-cigarettes are helpful to support quit smoking?’ with response categories of ‘helpful’, ‘not at all helpful’, and ‘not sure’. For analyses, we coded ‘not at all helpful’ and ‘not sure’ as ‘otherwise’. Addictiveness was measured by asking, ‘Do you believe that e-cigarettes are addictive?’ with response categories of ‘Yes’, ‘No’ and ‘Don't know’. Similarly, we grouped ‘no’ and ‘don't know’ as ‘otherwise’.

### Statistical analyses

Descriptive statistical analyses were conducted for sociodemographic variables and scores of perceptions of risk for smoking. Means and standard deviations (SD) were calculated for continuous data, while percentages were calculated for categorical data. Student’s t-test was used to compare the smoking risk perception difference between smokers and caregivers and between the participants who had different risk perceptions about e-cigarettes (e.g. e-cigarettes are less harmful than combustible cigarettes vs e-cigarettes are equally or more harmful than combustible cigarettes). The χ^2^ and Fisher’s exact probability tests were used to compare the perception of e-cigarettes between different sociodemographic groups. Logistic regression was used to determine predictors for e-cigarette harms, benefits, and addictiveness, adjusting for demographic and other characteristics. All collected data were analyzed with SAS 9.4 (SAS Institute, Inc., NC). The level of p<0.05 was considered statistically significant in all tests.

## RESULTS

Of the households approached, a total of 891/1142 (78%) in Taizhou and 320/356 (90%) in Dali participated in the survey (81% overall participation rate). Of the total (N=1211) household members who participated in the study, 668 were smokers and 543 were non-smokers. Of the participants (N=1211), a higher proportion (74%) was from the Taizhou region, the mean age was 44.2 (±13.54) years (range: 20–81), 58% were male, 34% were farmers and most (70%) had an education level of middle school. Almost all of the smokers were male (99%), while most of the non-smokers were female (97%); most participants were married (98%) (Table 1).

### Smoking and secondhand smoke exposure-related combined knowledge among participants

As shown in Table 2, non-smokers had significantly higher combined smoking and SHS exposure-related knowledge scores (3.97±0.86) (range: 1–5) than smokers (3.82±0.93) (p<0.01). Non-smoker’s knowledge scores were significantly higher in the items of ‘Smoking increases the risk of heart diseases’ (3.85±1.18 vs 3.65±1.34) and all items about SHS exposure-related knowledge. But no significant difference in the perceived risk of COPD and lung cancer were found between non-smokers and smokers (Table 2).

### Awareness and perceptions about e-cigarettes

As shown in Supplementary file Table S1, a higher proportion of smokers (57%) than non-smokers (47%) knew about e-cigarettes (p<0.01). Among those participants who knew about e-cigarettes (n=604), a significantly higher proportion of non-smokers (18%) than smokers (10%) thought e-cigarettes are addictive (p<0.01).

### Perceptions about e-cigarette harmfulness

As shown in Table 3, of those who knew about e-cigarettes, a significantly higher proportion of participants from Dali (77%) than Taizhou (59%) and those who were of ethnic minority (i.e. not Chinese Han) (76%) than Chinese Han (59%) thought e-cigarettes were less harmful (p<0.02). Participants who had a higher perceived risk (4.37±0.90) on the item ‘Smoking increases the risk of COPD’ thought e-cigarettes were less harmful than those who had a lower perceived risk (4.18±1.11) (p<0.05) (Table 4). Participants who had higher mean knowledge scores on SHS exposure-related items, including ‘Concerned about the harmful effects of SHS exposure to children’s health’, and ‘SHS exposure from smoker is harmful to adults' health’, also thought e-cigarettes were less harmful (Table 4).

**Table 3:** Association between demographic factors and perceptions of e-cigarette use in rural areas of Taizhou and Dali, China 2018

*Factors*	*Relative harmfulness (n=553)*			*Benefits (n=598)*			*Addictiveness (n=604)*		
	*Less harm n (%)*	*Equal or more harm n (%)*	*p*	*Helpful to quit smoking n (%)*	*Otherwise n (%)*	*p*	*Yes n (%)*	*Otherwise n (%)*	*p*
**Region**			**0.0086**			0.4906			0.7817
Taizhou	294 (59)	206 (41)		284 (56)	219 (44)		68 (13)	439 (87)	
Dali	41 (77)	12 (23)		50 (53)	45 (47)		12 (12)	85 (88)	
**Age** (years)			0.6787			0.3351			0.0608
≤34	162 (62)	99 (38)		169 (59)	119 (47)		35 (12)	254 (88)	
35–54	98 (59)	67 (41)		92 (52)	86 (48)		31 (17)	150 (83)	
≥55	62 (57)	46 (43)		63 (56)	50 (44)		9 (8)	105 (92)	
**Gender**			0.9047			0.1845			**0.0056**
Male	199 (60)	131 (40)		198 (54)	171 (46)		38 (10)	337 (90)	
Female	135 (61)	87 (39)		135 (59)	93 (41)		41 (19)	187 (81)	
**Ethnicity**			**0.0193**			0.4517			0.9858
Chinese Han	297 (59)	206 (41)		287 (57)	221 (43)		68 (13)	445 (87)	
Minority	38 (76)	12 (24)		47 (52)	43 (48)		12 (13)	79 (87)	
**Marital status**			0.3924*			0.8380*			0.8168*
Married	327 (61)	211 (39)		324 (56)	257 (44)		77 (13)	510 (87)	
Single	3 (38)	5 (62)		6 (60)	4 (40)		1 (10)	9 (90)	
Divorced or widowed	3 (60)	2 (40)		2 (40)	3 (60)		1 (20)	4 (80)	
**Occupation**			0.8311			0.7547			0.9599
Farmer	88 (61)	56 (39)		88 (52)	81 (48)		24 (14)	147 (86)	
Self-employed	71 (63)	41 (37)		70 (57)	52 (43)		16 (13)	108 (87)	
Unemployed	42 (55)	34 (45)		46 (61)	30 (39)		11 (14)	66 (86)	
Worker	76 (60)	51 (40)		74 (57)	55 (43)		15 (11)	118 (89)	
Other	57 (63)	34 (37)		55 (57)	42 (43)		12 (13)	82 (87)	
**Education level**			0.2058			0.3024			0.7182
Primary school or below	55 (53)	48 (47)		56 (54)	48 (46)		13 (12)	92 (88)	
Middle school	124 (64)	70 (36)		120 (53)	106 (47)		27 (12)	205 (88)	
High school or above	151 (61)	96 (39)		154 (60)	104 (40)		36 (14)	220 (86)	

* Fisher’s exact probability test.

**Table 4:** Association between smoking and SHS exposure related knowledge, and perceptions of e-cigarette use, rural areas of Taizhou and Dali, China 2018

*Variables*	*Relative harmfulness*			*Benefits*			*Addictiveness*		
	*Less harm mean±SD*	*Equal or more harm mean±SD*	*p*	*Helpful to quit smoking mean±SD*	*Otherwise mean±SD*	*p*	*Yes mean±SD*	*Otherwise mean±SD*	*p*
Smoking increases the risk of lung cancer	4.39±0.92	4.29±1.05	0.2782	4.49±0.87	4.14±1.11	**<0.0001**	4.35±0.96	4.33±1.01	0.8605
Smoking increases the risk of COPD	4.37±0.90	4.18±1.11	**0.0331**	4.41±0.90	4.16±1.06	**0.0027**	4.31±0.95	4.29±1.00	0.8766
Smoking increases the risk of heart diseases	4.03±1.15	3.86±1.18	0.1091	4.09±1.12	3.78±1.26	**0.0020**	3.96±1.20	3.95±1.19	0.9133
Concerned about the harmful effects of SHS exposure to children’s health	4.48±0.91	4.30±1.08	**0.0403**	4.52±0.89	4.30±1.07	**0.0074**	4.59±0.95	4.39±0.98	0.0967
SHS exposure from smokers can cause lung cancer in non-smokers	3.25±1.53	3.10±1.61	0.2711	3.33±1.50	2.99±1.68	**0.0105**	3.38±1.47	3.13±1.61	0.2106
SHS exposure from smoker is harmful to infant and children’s health	4.23±0.86	4.12±1.07	0.1830	4.28±0.87	4.10±1.04	**0.0189**	4.31±0.94	4.19±0.94	0.2657
SHS exposure from smoker is harmful to adults’ health	4.06±0.87	3.87±1.14	**0.0302**	4.10±0.91	3.81±1.13	**0.0010**	3.98±1.01	3.97±1.01	0.9621
Smoking around your children would cause adverse health effects among children	4.19±0.87	4.14±1.02	0.5751	4.24±0.87	4.12±1.00	0.1202	4.29±0.96	4.17±0.92	0.2811
Mean total score of smoking and SHS exposure related knowledge	4.15±0.66	4.03±0.81	0.0947	4.21±0.66	3.97±0.78	**0.0002**	4.19±0.64	4.08±0.73	0.2609

*p for Student’s t-tests.

### Perceptions about e-cigarette benefits

The mean total combined score of smoking and SHS exposure-related knowledge in participants who thought e-cigarettes are helpful to quit smoking was higher than in those who did not think e-cigarettes can help in quitting (p<0.05). Participants who thought smoking could increase the risk of lung cancer, COPD, or heart diseases, were more likely to believe that e-cigarettes were helpful to quit smoking than those who did not believe so (p<0.05 for all). Participants who had higher mean knowledge scores on SHS exposure-related items, including ‘Concerned about the harmful effects of SHS exposure to children's health’, ‘SHS exposure from smokers can cause lung cancer in non-smokers’, ‘SHS exposure from smoker is harmful to infant and children's health’ and ‘SHS exposure from smoker is harmful to adults' health’, were also inclined to believe that e-cigarettes were helpful to quit smoking (Table 4). No demographic variable was significantly associated with perceptions of e-cigarette benefits (Table 4).

### Perceptions about e-cigarette addictiveness

The only difference in the perceptions about e-cigarette addictiveness was found between different genders. A higher proportion of females (19%) than males (10%) perceived that e-cigarettes are addictive (p<0.01) (Table 3). None of the items about smoking and SHS exposure-related knowledge was significantly associated with perceptions of e-cigarette addictiveness (Table 4).

The multivariate logistic regression models including all the significant variables from Tables 3 and 4 (i.e. region, gender, ethnicity, risk of lung cancer, COPD and heart disease from smoking, risk of lung cancer from SHS exposure, and concerned about harmful effects of SHS to infant, children and adults’ health) and adjusting for region, sex, age, education level and occupation, identified only one variable associated with the benefits of e-cigarette use (i.e. e-cigarettes are helpful to quit smoking) that was associated with holding a higher knowledge about smoking and SHS exposure (OR=0.608; 95% CI: 0.450–0.820) (Table 5).

**Table 5:** Multivariate logistic regression analysis of perceptions of e-cigarette use among Chinese in rural areas of Taizhou and Dali (smokers and non-smoker)*

*Variables*	*Relative harmfulness (equal or more harm vs less harm)*		*Benefits (helpful to quit smoking vs otherwise)*		*Addictiveness (yes vs otherwise)*	
	*Smoker*	*Non-smoker*	Smoker***	*Non-smoker*	Smoker***	*Non-smoker*
	*OR (95% CI)*	*OR (95% CI)*	*OR (95% CI)*	*OR (95% CI)*	*OR (95% CI)*	*OR (95% CI)*
Knowledge about smoking and SHS exposure (total mean score)*	0.704 (0.514–0.965)	1.204 (0.707–2.051)	0.608 (0.450–0.820)	0.676 (0.403–1.135)	1.045 (0.624–1.751)	1.158 (0.589–2.278)
Knowledge about smoking and SHS exposure (total mean score)**	0.711 (0.520–0.973)	1.289 (0.761–2.184)	0.607 (0.450–0.819)	0.695 (0.417–1.156)	1.080 (0.645–1.808)	1.050 (0.546–2.016)

* Models were adjusted for region, sex, age, education level and occupation.

** Models were adjusted for region, age, education level and occupation.

*** Model is statistically significant (p<0.05).

## DISCUSSION

Findings from this study deepened our understanding of rural household adults’ self-reported usefulness and perceptions of e-cigarettes. The findings revealed distinct perceptions of e-cigarettes addictiveness among smokers and non-smoker caregivers as well as among men and women in the rural households with children. We only had three female smokers in our sample, which is obviously lower than the rural female smoking rate (2.2%, 95% CI: 1.5–3.2) as reported in the 2010 census^25^. Also, our small number of female smokers may be because our subjects were from households with children aged ≤5 years; many mothers might have quit smoking while pregnant or after giving birth.

We found that the awareness of e-cigarette use among our rural population sample is relatively high. Consistent with a previous study that found smokers (71.9%) than non-smokers (46.8%) had a much higher awareness of e-cigarettes^26^; our results showed that the awareness rates were 59% among smokers and 47% among non-smokers. It should be noted that, in this study, we only collected a positive or negative response of awareness of e-cigarettes instead of obtaining in-depth information about actual usage of e-cigarettes and how the awareness differs based on the status of e-cigarette use. Future studies should evaluate the relationships of e-cigarette awareness and perceptions with the actual usage of e-cigarettes. Also, our subjects are parents or household members with children at home. Having a child at home might have influenced participants’ perceptions and understanding about the hazards of cigarette smoking as well as e-cigarette use in consideration of the health of the child at home and the potential impact on the child to become a future cigarette smoker or e-cigarette user.

In an earlier study, 52.6% of the Chinese reported that e-cigarettes assist to quit smoking^26^. In the current study, we also found that over half of the participants perceived e-cigarettes as helpful in quitting smoking (56.1%) and less harmful (55.2%) relative to combustible cigarettes. Also, our results correspond to the summary findings from 18 Chinese e-cigarette websites that reported the following most common beneficial claims of e-cigarettes: health-related benefit (89%), no SHS exposure (78%), and helpful to quit smoking (67%)^27^. It can be expected that commercials of e-cigarettes introduce more positive perceptions of e-cigarettes, which may eventually lead to a higher prevalence of e-cigarette use. This influence might be particularly strong among smokers as they may be more sensitive to tobacco-related advertisements. However, very few e-cigarette products have been fully evaluated for their toxicological properties^28^, let alone in China where regulations over e-cigarettes are nearly non-existed so far. Although some studies have claimed possible benefits of using e-cigarettes^29^, they are not harmless and the long-term effects of their use are still unknown^30^. Considering the unregulated e-cigarette market in China and the lack of studies to assess the long-term impact of e-cigarettes on health, claims of health benefits and their effect on smoking cession should be postponed until these issues have been sufficiently studied. It is also critical to develop health campaigns to educate people about the facts of e-cigarette use, explaining what is known about the use of e-cigarettes as a possible smoking cessation tool and what are the potential health hazards. The World Health Organization also concluded that the evidence is insufficient to recommend e-cigarettes for quitting, and clinicians should direct patients to other cessation aids that are proven to be effective and safe^31^.

People tend to think e-cigarettes are less addictive than combustible cigarettes^32^. In this study, non-smokers had significantly higher awareness (38.7%) of the addictiveness of e-cigarettes than smokers (24%), though the general awareness was still very low. Although our knowledge of nicotine dependence from e-cigarettes is still limited^33^, nicotine in e-cigarettes may introduce addiction^34^.

### Strengths and limitations

This study contributes to the limited literature on the awareness of e-cigarette related harms, benefits, and addictiveness, among rural smokers and non-smokers in China. The survey provides insights that may inform future research directions and policy initiatives about the use and regulatory measures of e-cigarettes. There are five limitations in this study that warrant attention. First, the cross-sectional design of the study limits our ability to draw causal attributions from the research. Further research is needed to assess how beliefs about the risks and benefits of e-cigarettes among the adult household members change over time, and whether this change corresponds to changes in overall rates of smoking and e-cigarette use. Second, it is not clear whether participants’ perceptions towards e-cigarettes could be generalizable to other rural areas in China because both selected study areas are in southern China. However, our sample demographics included two diverse socioeconomic groups of Chinese rural smokers from an inland rural area (less affluent) and a coastal rural area (affluent), so we expect our findings might be generalizable to other regions of China that have populations with similar socioeconomic background. Moreover, the non-probabilistic nature of our sampling may also limit the generalizability/external validity of this study. Third, two measures of data collection (i.e. self-completion or interviewer completed) could introduce bias in the response. Fourth, our subjects might not represent the overall adult population in rural China, as our adult samples were from the households with children. While these population groups represent a section of the rural adults, their perceptions and behavior might be different from those households without a child. By having a child in the household and the possible intention to protect the child’s health, participants might have perceived more positive views towards tobacco and/or e-cigarette control. Fifth, although perceptions may be related to the future use of e-cigarettes, we did not test the link between perceptions and the actual usage of e-cigarettes in this study. The small number of current e-cigarette users in the current study did not allow us to examine factors such as motivations and the experience of using e-cigarettes that are more relevant to e-cigarette users. Instead, we documented a cross-sectional look at perceptions to assist policymakers in taking appropriate educational and policy measures to regulate e-cigarettes such as increasing education for students or limiting advertising under existing regulations.

## CONCLUSIONS

We found that about half of the rural households with a child at home are unaware of the harms that are associated with the use of e-cigarettes and believe that e-cigarettes can help smokers to quit smoking. Also, almost three-quarters of the participants did not consider e-cigarettes are addictive. There is a need for public health campaigns that will communicate the facts about e-cigarette use and disseminate evidence-based information about the potential harms of e-cigarettes and the lack of clear evidence surrounding the role of e-cigarettes as aids to quit smoking. As different factors were associated with supporting the use of e-cigarettes among smokers and non-smokers, public health campaigns about e-cigarette use may need to be tailored separately for these rural population groups. As knowledge about the harmfulness of smoking and SHS exposure was associated with perceived e-cigarette benefits, particular focus should be given to increasing knowledge about the health hazards related to smoking and SHS exposure.

## References

[1] McNeill A, Brose L, Calder R, Hitchman S, Hajek P, McRobbie H (2015). E-cigarettes: an evidence update: A report commissioned by Public Health England.

[2] Glantz SA (2018). Heated tobacco products: the example of IQOS. Tob Control.

[3] Grana R, Benowitz N, Glantz SA (2014). E-cigarettes: a scientific review. Circulation.

[4] Hartmann-Boyce J, McRobbie H, Bullen C, Begh R, Stead LF, Hajek P (2016). Electronic cigarettes for smoking cessation. Cochrane Database Syst Rev.

[5] Zhu SH, Zhuang YL, Wong S, Cummins SE, Tedeschi GJ (2017). E-cigarette use and associated changes in population smoking cessation: evidence from US current population surveys. BMJ.

[6] Villanti AC, Feirman SP, Niaura RS (2018). How do we determine the impact of e-cigarettes on cigarette smoking cessation or reduction? Review and recommendations for answering the research question with scientific rigor. Addiction.

[7] Kalkhoran S, Glantz SA (2016). E-cigarettes and smoking cessation in real-world and clinical settings: a systematic review and meta-analysis. Lancet Respir Med.

[8] El Dib R, Suzumura EA, Akl EA (2017). Electronic nicotine delivery systems and/or electronic non-nicotine delivery systems for tobacco smoking cessation or reduction: a systematic review and meta-analysis. BMJ Open.

[9] Zhao L, Mbulo L, Palipudi K, Wang J, King B (2019). Awareness and use of e-cigarettes among urban residents in China. Tob Induc Dis.

[10] Huang J, Duan Z, Wang Y, Redmon PB, Eriksen MP (2020). Use of Electronic Nicotine Delivery Systems (ENDS) in China: Evidence from Citywide Representative Surveys from Five Chinese Cities in 2018. Int J Environ Res Public Health.

[11] Barboza D (2014). China’s e-cigarette boom lacks oversight for safety.

[12] (2015). Chinese Center for Disease Control and Prevention - Tobacco Control Office. Chinese Adults Tobacco Survey Report 2015. Chinese Center for Disease Control and Prevention - Tobacco Control Office.

[13] Song AV, Morrell HE, Cornell JL (2009). Perceptions of smoking-related risks and benefits as predictors of adolescent smoking initiation. Am J Public Health.

[14] Tan AS, Bigman CA (2014). E-cigarette awareness and perceived harmfulness: prevalence and associations with smoking-cessation outcomes. Am J Prev Med.

[15] Giovenco DP, Lewis MJ, Delnevo CD (2014). Factors associated with e-cigarette use: a national population survey of current and former smokers. Am J Prev Med.

[16] Wang X, Zhang X, Xu X, Gao Y (2019). Perceptions and use of electronic cigarettes among young adults in China. Tob Induc Dis.

[17] Xiao L, Parascandola M, Wang C, Jiang Y (2019). Perception and current use of e-cigarettes among youth in China. Nicotine Tob Res.

[18] Tobacco Control Resource Center (2018). [International Tobacco Control Policy Assessment Project: ITC China Project Report - Findings from Wave 1 to Wave 5 (2006-2015)].

[19] Wang W, He Z, Feng N, Cai Y (2019). Electronic cigarette use in China: Awareness, prevalence and regulation. Tob Induc Dis.

[20] Chang R, Du L China Bans Online Sales of E-Cigarettes.

[21] Zhao D, Zuo Y, Urmi DS (2020). Perception of E-cigarette Use among Adult Users in China: A Mixed-method Study. Int J Environ Res Public Health.

[22] (2017). [Statistical Bulletin of National Economic and Social Development of Taizhou City in 2017].

[23] (2017). [Statistical Bulletin of National Economic and Social Development of Dali Bai Autonomous Prefecture in 2017].

[24] [Statistical Bulletin of National Economic and Social Development of Jianchuan County in 2016].

[25] Li Q, Hsia J, Yang G (2011). Prevalence of smoking in China in 2010. N Engl J Med.

[26] Xu Y, Guo Y, Liu K, Liu Z, Wang X (2016). E-cigarette awareness, use, and harm perception among adults: a meta-analysis of observational studies. PLoS One.

[27] Yao T, Jiang N, Grana R, Ling PM, Glantz SA (2016). A content analysis of electronic cigarette manufacturer websites in China. Tob Control.

[28] Orr MS (2014). Electronic cigarettes in the USA: a summary of available toxicology data and suggestions for the future. Tob Control.

[29] Nutt DJ, Phillips LD, Balfour D (2014). Estimating the harms of nicotine-containing products using the MCDA approach. Eur Addict Res.

[30] National Center for Chronic Disease Prevention and Health Promotion - US Office on Smoking and Health (2016). E-Cigarette Use Among Youth and Young Adults: A Report of the Surgeon General.

[31] WHO Framework Convention on Tobacco Control (2014). Electronic nicotine delivery systems: Report by WHO. https://apps.who.int/iris/bitstream/handle/10665/147110/FCTC_COP6_10Rev1-en.pdf?sequence=1&isAllowed=y.

[32] Choi K, Forster J (2013). Characteristics associated with awareness, perceptions, and use of electronic nicotine delivery systems among young US Midwestern adults. Am J Public Health.

[33] Schroeder MJ, Hoffman AC (2014). Electronic cigarettes and nicotine clinical pharmacology. Tob Control.

[34] Liu G, Wasserman E, Kong L, Foulds J (2017). A comparison of nicotine dependence among exclusive E-cigarette and cigarette users in the PATH study. Prev Med.

