# Supplementary material for: Perceptions of e-cigarettes among smokers and non-smokers in households with children in rural China: A cross-sectional study
Source: Tob Induc Dis. 2021 Apr 9;19:25. doi: 10.18332/tid/133264 (PMC8033598; doi:10.18332/tid/133264)
Supplement: Supplementary file 1 [file TID-19-25-s1.pdf]

Supplementary file: Perceptions about e-cigarettes harmfulness, benefits and addictiveness by smoking status; rural areas of Taizhou and Dali, China 2018 (N=1161).

	Overall n (%)	Smoker n (%)	Nonsmoker n (%)	P-value
**Heard about e-cigarettes (n=1161)**				**0.0013**
Yes	613(53)	375(57)	238(47)	
No	548(47)	284(43)	264(53)	
**e-cigarettes relative harmfulness (n=553)* [a]**				0.8981
Less harm	324(59)	197(59)	127(58)	
Equal or more harm	229(41)	138(41)	91(42)	
**e-cigarettes benefits (n=598)* [a]**				0.0958
Helpful to quit smoking	334(56)	194(53)	140(60)	
Otherwise	264(44)	171(47)	93(40)	
**e-cigarettes addictiveness (n=604)*[a]**				**0.0035**
Yes	80(13)	37(10)	43(18)	
Otherwise	524(87)	332(90)	192(82)	

*Applicable only to those who has heard about e-cigarettes; [a] total number not equal to 613 due to missing cases.

© 2021 Zhao D. et al
